# Radiographic analysis of the proximal femoral anatomy in the Croatian population

**DOI:** 10.1007/s00264-021-04942-5

**Published:** 2021-01-13

**Authors:** Hrvoje Mokrovic, Simona Komen, Leo Gulan, Gordan Gulan

**Affiliations:** 1 Clinic of Orthopaedic Surgery Lovran, M.Tita 1, 51415 Lovran, Croatia; 2 Department of Traumatology, Clinical Hospital Center Rijeka, Kresimirova 42, 51000 Rijeka, Croatia; 3 Department of Anatomy, Faculty of Medicine University of Rijeka, B. Branchetta 20, Rijeka, Croatia; 4 Department of Orthopaedic and Physical Medicine, Faculty of Medicine University of Rijeka, B. Branchetta 20, Rijeka, Croatia

**Keywords:** Proximal femoral anatomy, Croatian population, Femoral offset

## Abstract

**Purpose:**

The goal of total hip endoprosthesis is to achieve painless and functional hip for long term. Accurate reconstruction of hip anatomy largely depends on the implant design. In order to select an implant in correspondence with the native hip, the proximal femoral morphology has been in focus of many studies in the past years. The purpose of this study is to analyze proximal femoral geometry in the Croatian population by radiographic evaluation.

**Methods:**

We conducted a retrospective study analyzing conventional radiographies of the hip, obtained within the last four years from the database of Clinic for Orthopaedic Surgery Lovran. The number of studied patients was 300,168 women and 132 men. The proximal femoral geometric parameters assessed were as follows: femoral head diameter, femoral neck length, neck-shaft angle, angle of femoral neck anteversion, and lateral femoral offset. The results obtained were compared between genders and with results of other studies.

**Results:**

Proximal femoral anatomy differed in femoral head diameter and lateral femoral offset between males and females in our group of patients, while femoral neck length, femoral neck shaft angle, and femoral neck anteversion have shown similar values in both genders. Our study also showed specificity of the Croatian population in almost all parameters of proximal femoral anatomy, in comparison with other ethnic groups.

**Conclusion:**

Our results support the observation on high diversity in the morphology of the proximal femur and the specificity of the proximal femoral anatomy of the Croatian population.

## Introduction

Frequency of total hip arthroplasty (THA), as effective treatment for end stage of hip osteoarthritis, has been increased all over the world [1]. The goal of THA is to achieve painless and functional hip for long period. Many factors influence the longevity of THA, such as implant design, material type, body weight, surgical technique, and hip anatomy reconstruction [2]. Multiple authors in their studies described the effects of incorrectly reconstructed hip anatomy with THA, resulting in patients’ dissatisfaction, leg length inequality, limping, pain, increased material wear, and loosening of hip prosthesis [3], and in many of this cases, revision of total hip replacement is required. Accurate reconstruction of hip anatomy grossly depends on implant design [4]. In order to select an implant in correspondence with the native hip, the proximal femoral morphology has been in focus of many studies in the past years. Studies have shown significant differences in the anatomy of the proximal femur between races, ethnic groups, and genders but also between geographic regions of the same population [5]. Therefore, these studies established the need for developing ethnic- [6] and gender-specific implants [7]. Reviewing the literature, we did not find too many data on the hip anatomy of the southeast Europe population. The subject of this study is to analyze proximal femoral geometry by radiographic evaluation in the Croatian population, which geographically belongs to the mentioned part of Europe.

## Patients and methods

**Fig. 1:**
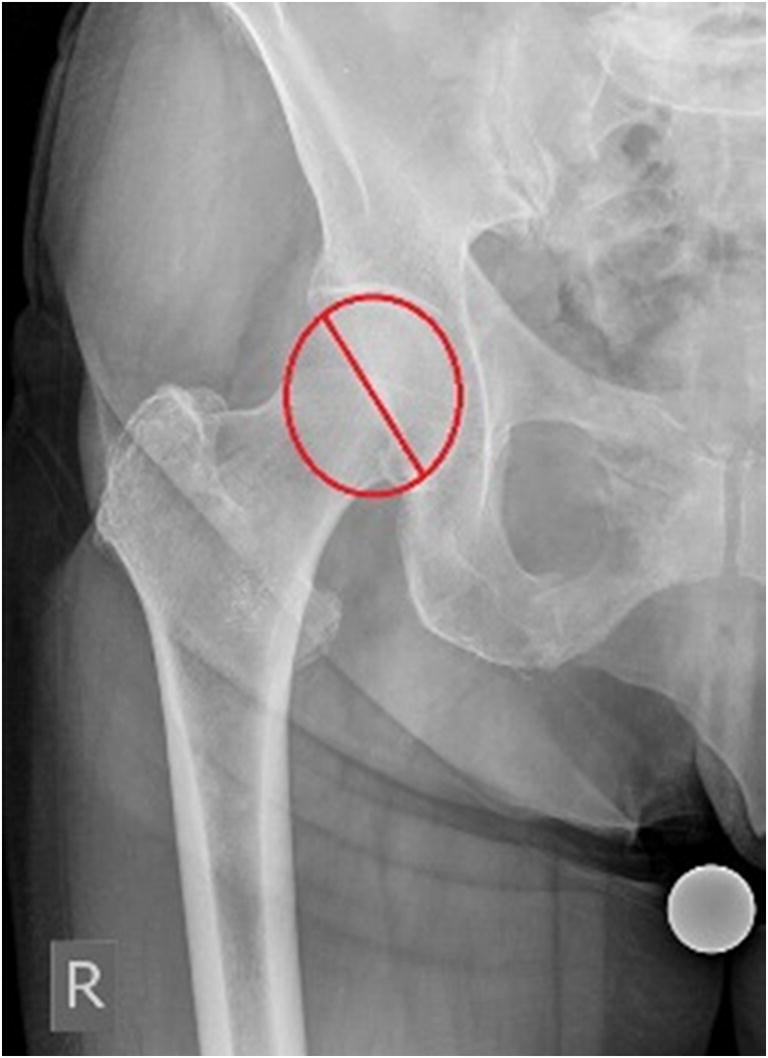
Femoral head diameter is the diameter of a complete circle drown around the femoral head

**Fig. 2:**
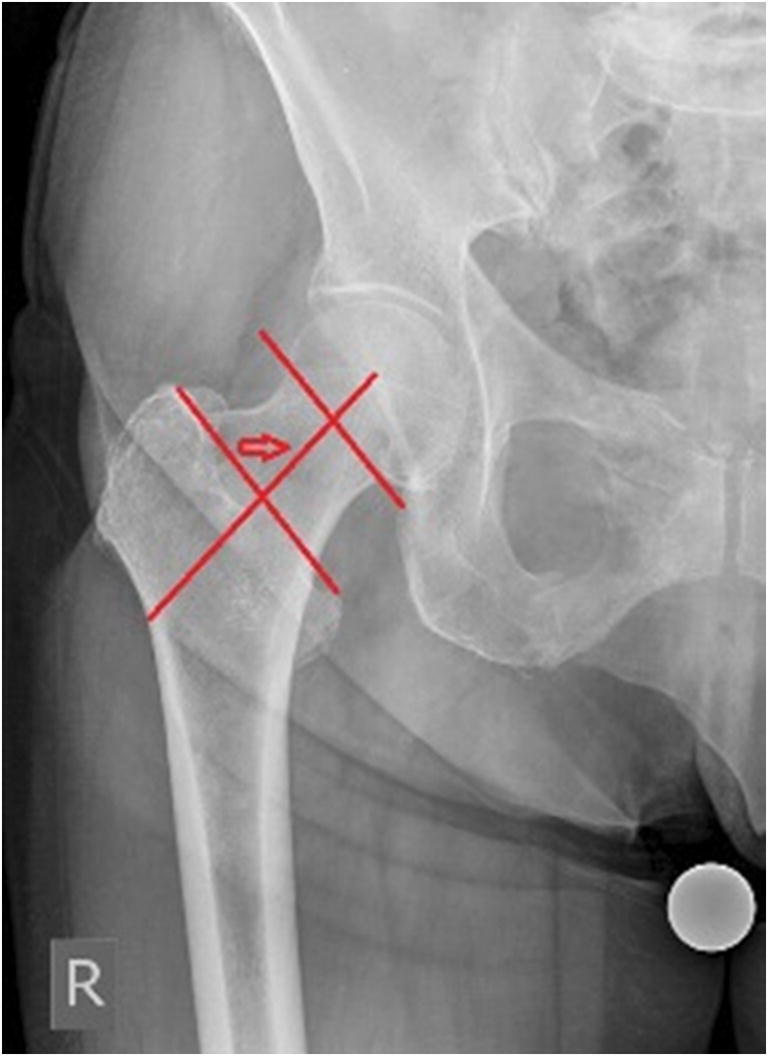
Femoral neck length is the distance between the lateral margin of the femoral head and the superior base of the trochanteric region marked with arrow

**Fig. 3:**
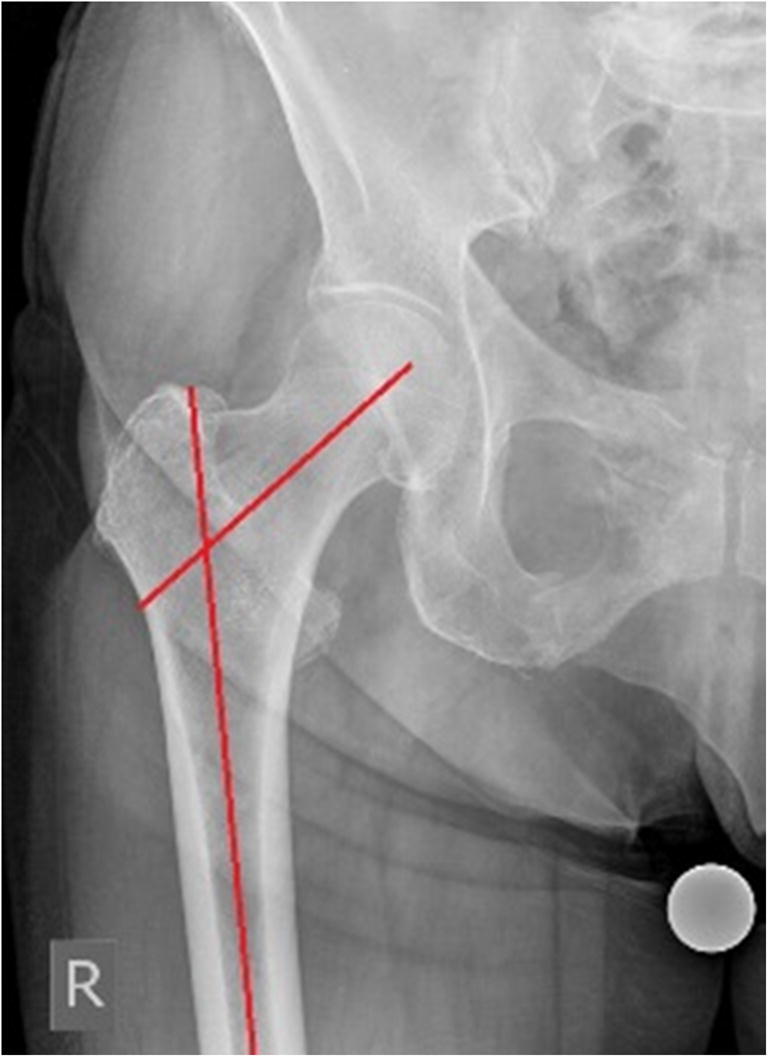
Femoral neck shaft angle is angle formed by the intersection of the neck axis line and the femoral shaft anatomical axis line

**Fig. 4:**
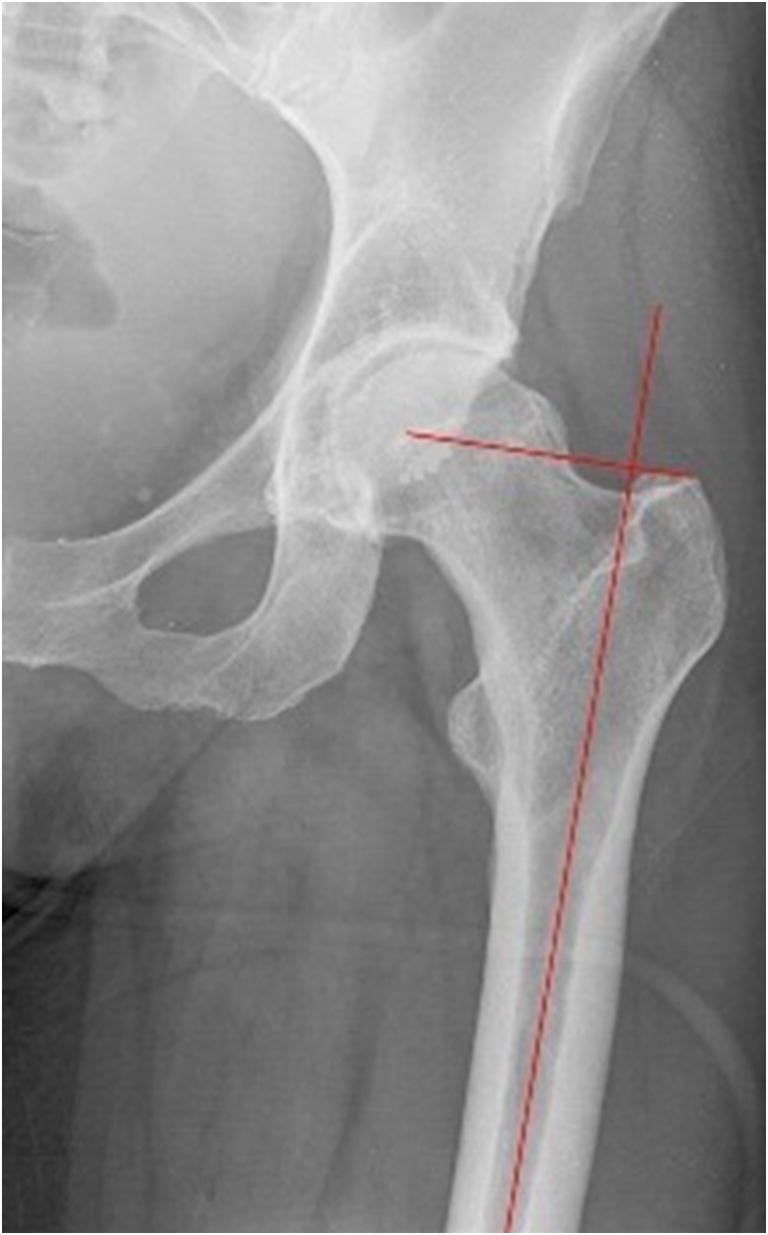
Lateral femoral offset is the distance between the femoral head centre of rotation and the midline of long axis of the femur

We conducted a retrospective study analyzing the antero-posterior (AP) and axial radiographies of the hip, obtained within the last four years from the database of the Clinic for Orthopaedic Surgery Lovran. This study was approved by the ethical committee. The number of studied patients was 300, 168 women and 132 men. Average age of analyzed patients was 64.28 ± 13.17 (women 62.80 ± 14.72, men 66.16 ± 10.76). We excluded from this study patients with hip disorders, previous hip surgery, hip fracture history, and any infectious lesion, and OA changes grade IV according to Kellgren-Lawrence. AP view was obtained by standard pelvis positioning protocol for hip arthroplasty with the beam of the X-ray directed toward the midline above the symphysis pubis and with both lower extremities in 15° of internal rotation. The axial view of the hip was taken with the patient in the supine position, the image receptor placed superior to the iliac crest and angled approximately 20–45° to match the angle of the femoral neck, the central ray angled to be perpendicular to the long axis of the femoral neck, the centering point 13 cm distal to the neck of the femur, and patient’s unaffected hip flexed and abducted. Radiological measurements of proximal femoral geometric parameters were performed using the Agfa IMPAX Orthopaedic Tools program. The proximal femoral geometric parameters assessed were as follows: femoral head diameter, neck-shaft angle, angle of femoral neck anteversion, and lateral femoral offset (Figs. 1, 2, 3, and 4).

1. Femoral head diameter (FHD) is the diameter of a complete circle drawn around the femoral head.
2. The femoral neck length (FNL) is the distance between the lateral margin of the femoral head and the superior base of the trochanteric region**.**
3. The neck-shaft angle (FNSA) is the angle formed by the intersection of the neck axis line and the femoral shaft anatomical axis line.
4. Femoral neck anteversion (FNA) is the anterior inclination of the femoral neck in relation to the transcondylar knee axis projected on a plane perpendicular to the shaft axis. The angle of femoral neck anteversion was measured using biplane roentgenographic examination of the femur according to the procedure described by Magilligan [8].
5. Lateral femoral offset (LFO**)** is calculated as the distance from the center of rotation of the femoral head to the midline of the long axis of the femur**.** There are several methods for establishing the center of hip rotation. We used Pierchon’s method [9], where the radiographic image of the teardrop is used as a reference point to determine the center of rotation.

The parameters obtained were compared between males and females and statistically analyzed. We also compared results from our study with results from similar studies for various ethnic groups. Statistical analysis was performed using Statistica 13.1 (Dell Inc. (2016). version 13, Tulsa, USA). The Kolmogorov-Smirnov test was applied to test the normality of distribution. Continuous variables with normal distribution were expressed as mean ± standard deviation (SD). The differences between two groups were determined by the unpaired Student test, and *P* values lower than 0.05 were considered as significant.

## Results

The results of radiographic measurements of proximal femoral geometric parameters in Croatian population are presented in Table 1. We compared proximal femoral geometry mean values of both genders. Results are presented in the Table 2*.* Analyzing the data revealed that mean values of female femoral head diameter and lateral femoral offset were significantly smaller than the male values of same parameters. There was no statistical significance of mean values in other femoral geometric parameters between genders.

**Table 1:** Results of radiological measurements of proximal femoral geometric parameters in the Croatian population

Parameters	Mean ± SD (min.–max.)
Femoral head diameter/mm	38.84 ± 5.32 (30.00–52.10)
Femoral neck length/mm	44.29 ± 4.31 (32.60–52.10)
Neck shaft angle/°	125.34 ± 4.26 (115.60–140.70)
Angle of femoral neck anteversion/°	16.53 ± 1.97 (11.10–21.10)
Lateral femoral offset/mm	51.22 ± 8.44 (42.30–60.00)

**Table 2:** Comparison of the proximal femoral parameters between genders in Croatian population

Parameters Mean ± SD (min.–max.)	Female in general	Male in general	*t* value	*P* value between male and female
Femoral head diameter/mm	37.34 ± 5.18 (30.00–52.10)	40.74 ± 4.92 (30.30–52.10)	− 3.33	0.001
Femoral neck length/mm	44.04 ± 4.95 (32.60–52.10)	44.60 ± 3.36 (40.30–52.10)	− 0.64	0.526
Neck shaft angle/°	125.50 ± 5.10 (115.60–140.70)	125.13 ± 2.91 (116.80–134.00)	0.44	0.662
Angle of femoral neck anteversion/°	16.78 ± 1.86 (10.30−21.10)	16.34 ± 1.85 (10.10–17.30)	0.56	0.576
Lateral femoral offset/mm	49.44 ± 4.88 (42.30–57.30)	53.48 ± 11.14 (42.30–60.10)	− 2.43	0.016

### Femoral head diameter

The obtained mean value of the femoral head diameter in our study was 38.84 ± 5.32 mm, with values ranging from 30.00 to 52.10 mm. The mean value of femoral head diameter among females was 37.34 ± 5.18 and 40.74 ± 4.92 mm among males. There is a statistically significant difference of femoral head diameter mean values between genders (*P* = 0.001).

### The femoral neck length

The mean value of femoral neck length was 44.29 ± 4.31 mm, with values ranging from 32.60 to 52.10 mm. The mean value of femoral neck length among females was 44.04 ± 4.95 mm and 44.60 ± 3.36 mm among males. There is no statistically significant difference in the mean value of femoral neck length between the genders.

### The neck-shaft angle

The mean value of neck-shaft angle was 125.34 ± 4.26°, with values ranging from 115.60° to 140.70°. The mean value of the neck-shaft angle among females was 125.50 ± 5.10° and 125.13 ± 2.91° among males. There is no statistically significant difference in the mean values of neck-shaft angle between genders.

### Angle of femoral neck anteversion

The mean value of femoral neck anteversion was 16.53 ± 1.97 °, with values ranging from 11.10 to 21.10°. The mean value of femoral neck anteversion among females was 16.78 ± 1.86° and 16.34 ± 1.85° among males. There is no statistically significant difference in the mean values of femoral neck anteversion between genders.

### Lateral femoral offset

The mean value of the lateral femoral offset was 51.22 ± 8.44 mm, with values ranging from 42.30 to 60.10 mm. The mean value of lateral femoral offset among females was 49.44 ± 4.88 mm and 53.48 ± 11.14 mm among males, which was statistically significant difference (*P* = 0.016).

## Discussion

Multiple studies analyzed the proximal femoral morphology using different specimens and methods of measuring [10, 11]. We decided to analyze conventional radiographies since they are used as a standard in pre-operative planning for total hip arthroplasty. Proximal femoral anatomy became very important, because reconstruction of the native individual values was recognized as a prerequisite factor for the success in total hip arthroplasty [12]. Hip anatomy is a subject to a high individual variability [13]. Gender is one of the parameters associated with anatomical hip variability [14]. In our group of patients, we observed statistically significant differences between genders in FHD and LFO parameters, while FNA and FNSA were of similar values in both genders. Many studies observed differences in femoral geometry between races and ethnic group [5, 6]. We compared values from this study with values from similar studies which described proximal femoral anatomy of different races and ethnic groups, but we also compared our results with the results of authors who analyzed Caucasian proximal femoral geometry [15–19]. We detected differences in proximal femoral geometry between Croatian population and other ethnic groups. The comparison of the mean values of proximal femoral geometry between Croatian population and various ethnic groups is shown in Table 3. Comparing our results of proximal femoral geometry with the Asian population from Korea [20] and China [21], we observed that Croatian population has significantly smaller FNSA and FNA but higher LFO. Comparing the results of the Croatian population with the results of Indian studies, proximal femoral geometry has shown similarities in FNSA with the results of Rawal et al. [6] but smaller values in comparison with the study of Minakshi et al. [22]. The results for FHD are smaller and for LFO higher in our study compared with both of these studies. FNA is higher in the Croatian population in comparison with the findings by Rawal et al. [6]. Comparing our results with results from varius studies of the Caucasian population, we also detected some differences in proximal femoral geometry. The FHD in our study was 38.84 ± 5.32 mm, while the median value of the femoral head diameter in the study of Rubin et al. [16] was 43.4 ± 2.6 mm. Unnanuntana et al. [19] analyzed proximal femoral morphology in American Caucasians, and the diameter of the femoral head in his study was 52.09 ± 4.4 mm, significantly larger than in the Croatian population. With regard to FNSA, varying ranges have been described as reference ranges. Boese et al. reported the value ranging from 98 to 160° in the healthy population [13]. Normal range of the FNSA is generally considered between 120 and 140° [23] with a global mean of 126.4° [24]. Values < 120° are classified as coxa vara and > 140° as coxa valga [25]. FNSA together with femoral neck length directly affects the LFO. The reconstruction of LFO largely depends on femoral stem design. Offset reduction of more than 15% or more than 5 mm in comparison with native value reduces the abductor moment arm influencing the gate pattern [26]. The FNSA in our study was 125.34 ± 4.26°, and this is significantly lower in comparison with that of the Turkish [15] 129.71 ± 4.4**°**, French 129.2 ± 7.8**°** [17], and Norwegian population [18] 127.7 ± 7.6**°** but higher than in the Swiss population [16] 122.9 ± 7.6. The mean value for LFO in the Croatian population is 51.22 ± 8.44 mm. This was the highest value in comparison with the values reported in all analyzed studies regardless of race or ethnicity, and the difference was statistically significant. Another parameter of proximal femoral anatomy that should be reconstructed during hip arthroplasty is FNA. Error in adjusting the version of the femoral component of endoprosthesis will modify the lever arms, foot position, and the gait pattern and is recognized as a risk factor for hip dislocation [27] and can decrease periprosthetic bone density [28]. The literature revealed a discrepancy between native femoral neck anteversion and version of the femoral component of endoprosthesis, ranging in excessive anteversion to retroversion, especially in cementless prostheses [29]. In most studies, the degree of version of the femoral component was significantly increased compared to the degree of native femoral neck anteversion [30]. Previous studies have shown that femoral anteversion of Asians is generally larger than that of Caucasians where the mean value is about 10° [12]. FNA in the Croatian population is 16.53 ± 1.97°, between Asian and Caucasian values.

**Table 3:** Comparative analysis of the proximal femoral geometric parameters reported in different studies; the results significantly statistically different (*P* < 0.05) from the results obtained from present study for Croatian population are asterisk marked

Parameters Mean ± SD	Croatian population	Rawal [6] (Indian) *N* = 98	Unnanuntana [19] (Americans Caucasian) *N* = 200	Acar [15] (Turkish) *N* = 380	Rubin [16] (Swiss) *N* = 32	Husmann [17] (French) *N* = 300	Reikerås [18] (Norwegian) *N* = 48	Cho [20] (Korean) *N* = 202	Lin [21] (Chinese) *N* = 100	Minakshi (22) (Indian) *N* = 91
Femoral head diameter/mm	38.84 ± 5.32	45.41 ± 3.7* *P* < 0.001	52.09 ± 4.4 *P* < 0.001	47.13 ± 3.4* *P* < 0.001	43.4 ± 2.6* *P* < 0.001	–	–	45.50 ± 3.4* *P* < 0.001	–	42.32 ± 4.1* *P* < 0.001
Femoral neck length/mm	44.29 ± 4.31	48.4 ± 5.6* *P* < 0.001	–	34.56 ± 4.7* *P* < 0.001	47 ± 7.2* *P* = 0.01	–	–	–	45.40 ± 3.2	44.75 ± 8
Neck shaft angle/°	125.34 ± 4.26	124.42 ± 5.5	132.69 ± 5.9* *P* < 0.001	129.71 ± 4.4* *P* < 0.001	122.9 ± 7.6* *P* = 0.024	129.2 ± 7.8* *P* < 0.001	127,7 ± 7,6* *P* = 0.017	130.27 ± 5.4* *P* < 0.001	129.88 ± 5.7* *P* < 0.001	128.90 ± 4.5* *P* < 0.001
Angle of femoral neck anteversion/°	16.53 ± 1.97	10.9 ± 4.2* *P* < 0.001	–	–	–	–	10.4 ± 6.7* *P* < 0.001	–	21.58 ± 3.3* *P* < 0.001	–
Lateral femoral offset/mm	51.22 ± 8.44	40.23 ± 4.8* *P* < 0.001	41.16 ± 6.0* *P* < 0.001	41.11 ± 5.3* *P* < 0.001	–	40.5 ± 7.5* *P* < 0.001	–	37.88 ± 5.4* *P* < 0.001	–	42.92 ± 5.5 * *P* < 0.001

## Conclusion

Our results support the observations from similar studies on high diversity in the morphology of the proximal femur, not only between racial and ethnic groups but also depending on the geographic regions of the same population. Compared with other ethnic groups, our study showed specificity of the Croatian population in most parameters of proximal femoral anatomy. We hope that our results will improve understanding of proximal femur morphology and may help to choose implant in correspondence with the anatomy of the hip for the majority of our population.

## Data Availability

The data generated and analyzed during the current study are available from the corresponding author on reasonable request.
